# Unresolved questions in selection of therapies for treatment-naïve chronic lymphocytic leukemia

**DOI:** 10.1186/s13045-023-01469-7

**Published:** 2023-07-08

**Authors:** Rory Bennett, Mary Ann Anderson, John F. Seymour

**Affiliations:** 1 Department of Clinical Haematology, Royal Melbourne Hospital and Peter MacCallum Cancer Centre, 305 Grattan St, Parkville, Melbourne, VIC 3000 Australia; 2 Division of Blood Cells and Blood Cancer, The Walter and Eliza Hall Institute, 1G, Royal Parade, Parkville, Melbourne, VIC 3052 Australia; 3 University of Melbourne, Grattan St, Parkville, Melbourne, VIC 3010 Australia

**Keywords:** CLL, Frontline therapy, Sequencing, BTK inhibitor, BCL2 inhibitor, Chemoimmunotherapy

## Abstract

**Background:**

The treatment landscape for chronic lymphocytic leukemia (CLL) continues to undergo considerable evolution. Optimal selection of initial therapy from multiple effective options provides a major challenge for clinicians, who need to consider both disease and patient factors in conjunction with a view to sequencing available therapies in event of disease relapse.

**Review:**

We explore the most topical clinically relevant unresolved questions through discussion of important available pertinent literature and propose expert opinion based on these data. (1) Shrinking role of chemoimmunotherapy (CIT); while novel therapies are generally superior, we highlight the utility of FCR for IGHV-mutated CLL. (2) Choosing between inhibitors of Bruton’s tyrosine kinase (BTKi); while efficacy between agents is likely similar there are important differences in toxicity profiles, including the incidence of cardiac arrhythmia and hypertension. (3) BTKi with or without anti-CD20 monoclonal antibodies (mAb); while obinutuzumab-acalabrutinib (AO) may confer superior progression-free survival to acalabrutinib (Acala), this is not true of rituximab (Ritux) to ibrutinib (Ib)—we highlight that potential for increased side effects should be carefully considered. (4) Continuous BTKi versus time-limited venetoclax-obinutuzumab (VenO); we propose that venetoclax (Ven)-based therapy is generally preferable to BTKi with exception of *TP53* aberrant disease. (5) BTKi-Ven versus VenO as preferred time-limited therapy; we discuss comparable efficacies and the concerns about simultaneous 1L exposure to both BTKi and Ven drug classes. (6) Utility of triplet therapy (BTKi-Ven-antiCD20 mAb) versus VenO; similar rates of complete response are observed yet with greater potential for adverse events. (7) Optimal therapy for *TP53* aberrant CLL; while limited data are available, there are likely effective novel therapy combinations for *TP53* aberrant disease including BTKi, BTKi-Ven ± antiCD20 mAb.

**Conclusion:**

Frontline therapy for CLL should be selected based on efficacy considering the patient specific biologic profile of their disease and potential toxicities, considering patient comorbidities and preferences. With the present paradigm of sequencing effective agents, 1L combinations of novel therapies should be used with caution in view of potential adverse events and theoretical resistance mechanism concerns in the absence of compelling randomized data to support augmented efficacy.

## Background

Preferred first-line (1L) therapy for chronic lymphocytic leukemia (CLL) has evolved considerably over the last decade. Targeted inhibitors of Bruton tyrosine kinase (BTKi) and the anti-apoptotic protein B-cell lymphoma 2 (BCL2i—the most advanced example in development being venetoclax [Ven]) are highly effective first-line strategies for treatment of CLL with significant activity demonstrated across all genomic risk subgroups. Both continuous BTKi [1–6] and fixed-duration Ven in combination with obinutuzumab (Obi), (VenO) [7–10] demonstrate superior progression-free survival (PFS) and/or overall survival (OS) compared with chemoimmunotherapy (CIT) as initial therapies, regardless of patient fitness. Treatment guidelines from the National Comprehensive Cancer Network (NCCN, updated 2023) [11] and European Society of Medical Oncology (ESMO, 2021) [12] for treatment-naïve CLL endorse-targeted therapy as first therapy for both high-risk and non-high-risk patients, while the role of CIT continues to shrink.

Despite these advances, several key questions remain unanswered including:

1. Considering the role, if any, for CIT in 1L treatment of CLL,
2. How best to combine and utilize targeted agents in 1L therapy, and
3. The impact of genomic profile on choice of targeted agent

While recommended thresholds for treatment initiation per iwCLL were established in the CIT-era, no data support earlier treatment with either CIT or novel agents [13, 14]. Selection of initial therapy for individual patients must consider efficacy, toxicities, long-term impacts on subsequent therapy options and patient preference while ensuring equity of access with increasingly costly treatments. Although selection of 1L therapy should be individualized, patient fitness must be considered. The majority of patients with CLL are considered to be ‘unfit’ due to advanced age or pre-existing comorbidities. While patient frailty may be identified across multiple domains by geriatric assessment tools [15, 16], contemporaneous studies of novel agents have generally defined an ‘unfit’ patient as those > 65 years of age, or with Cumulative Illness Rating Scale (CIRS) ≥ 6, and/or creatinine clearance of < 70 ml/min.

While the utility of drug classes such as ROR1 antibodies, MCL1 inhibitors, BTK protein degraders, and immunotherapies (bispecific antibodies, chimeric antigen receptor [CAR] T-cell therapy) are being explored in relapsed/refractory (R/R) CLL, none are expected to imminently impact 1L therapeutic selection [17].

**Fig. 1:**
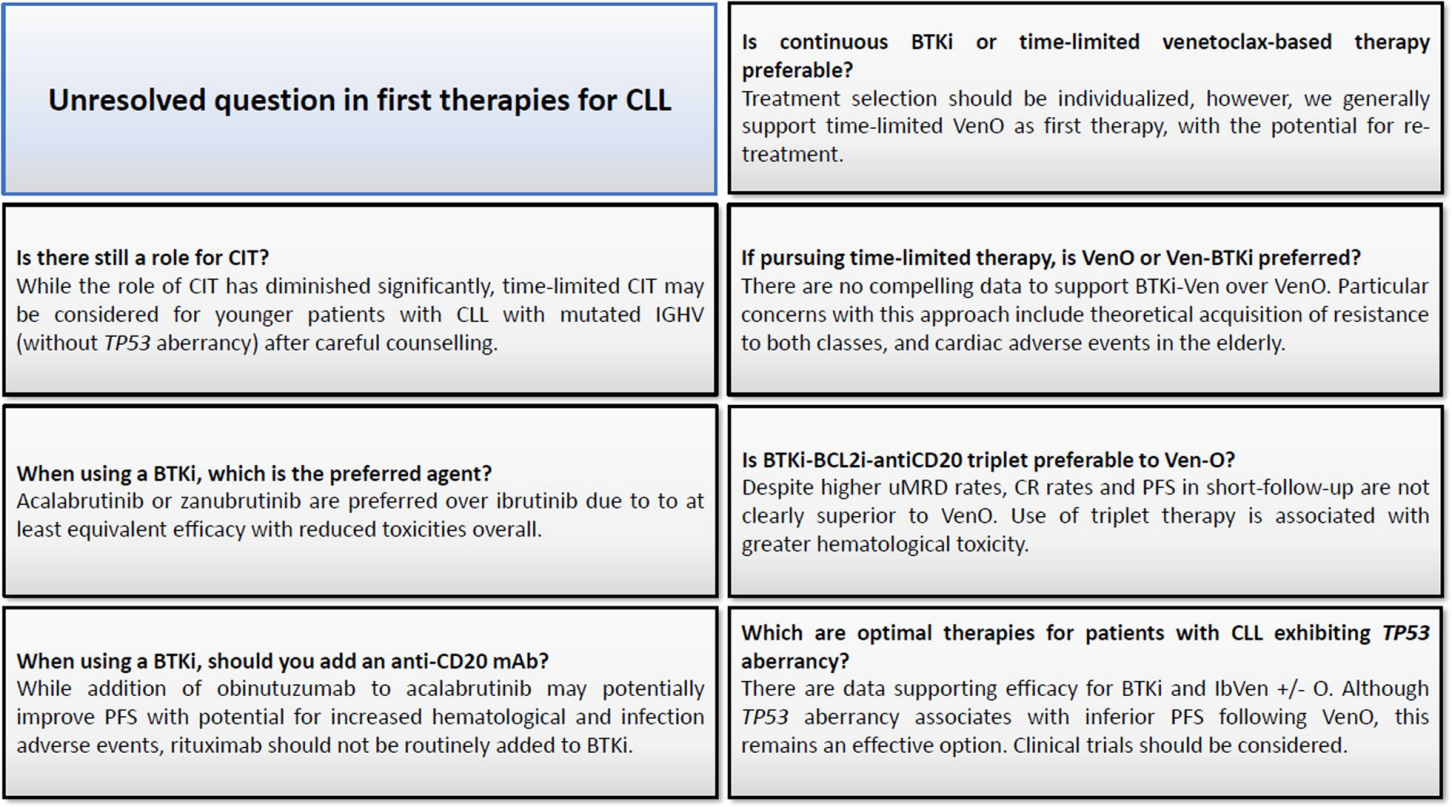
Graphical summary of unresolved questions in first therapies for CLL. BTKi—Bruton’s tyrosine kinase inhibitor; BTKi-Ven—Bruton’s tyrosine kinase inhibitor-venetoclax combination; CIT—chemoimmunotherapy; CR—complete response; Ib-Ven ( ±) O—ibrutinib-venetoclax ± obinutuzumab; mAb—monoclonal antibody; PFS—progression-free survival; uMRD—undetectable measurable residual disease; VenO—venetoclax-obinutuzumab

In this review, we explore a number of unresolved questions regarding optimal 1L therapy for CLL. A graphical summary is provided, see Fig. 1.

## Is there still a role for chemoimmunotherapy as first therapy?

While CIT is unsuitable for the majority of patients, it may be considered for carefully selected individuals without genomic adverse-risk disease.

Fludarabine-cyclophosphamide-rituximab (FCR) and bendamustine-rituximab (BR) are previously established standards of care CIT combinations for fit and unfit patients, respectively, with an indication for treatment, without del(17p) and/or *TP53* mutation (*TP53* aberrancy) [18, 19]. In the German CLL8 study, FCR demonstrated superior PFS and OS compared with FC after median follow-up of 5.9 years, see Table 1 [18, 20]. In the CLL10 trial from the same study group, FCR displayed superior PFS compared with BR for young patients (< 65 years); however, in *post-hoc* analysis no significant difference was observed for patients ≥ 65 years (median 57.9 vs. 48.5 months, HR 1.352 [95% CI 0.912–2.006], *p* = 0.134) for whom incidence of adverse events was higher, including hematological toxicities [19, 21].

**Table 1:** Main efficacy outcomes of pivotal clinical trials in untreated CLL

Pivotal frontline CLL studies	ORR/CR + CRi	Best uMRD/timepoint otherwise stated	PFS	OS
CLL8 [18, 20, 127] Phase III, fit patients (aged 30–81) FCR vs. FC—intended six cycles each	90%/44% (FCR) vs. 80%/22% (FC)	Final re-staging: [PB] 63% (FCR) vs. 35% (FC), (*p* < 0.0001)	Median follow-up 5.9 years; median PFS 56.8 m (FCR) vs. 32.9 m (FC), HR 0.59 (95% CI 0.50–0.69), (*p* < 0.001)	Median follow-up 5.9 years; OS rates 87% vs. 83% (FC), (*p* = 0.012)
CLL10 [18, 21] Phase III, fit patients without del(17p) BR vs. FCR—intended six cycles each	97.8%/31.5% (BR) vs. 97.8%/40.7% (FCR)	Final re-staging: [PB] 62.9% (BR) vs. 74.1% (FCR) [BM] 31.6% (BR) vs. 58.1% (FCR)	Median follow-up 58.2 months; median PFS 42.3 m (BR) vs. 57.6 m (FCR), HR 1.593 (95% CI 1.271–1.996), (*p* < 0.0001)	Median follow-up 58.2 months; OS rates 80.1% (BR) vs. 80.9% (FCR), HR 1.108 (95% CI 0.755–1.627), (*p* = 0.599)
RESONATE-2 [45, 128] Phase III, patients ≥ 65y without del(17p) Ib vs. Chl, cross-over allowed upon PD Ib—until PD or intolerance, Chl—up to 12 cycles	Initial report: 86%/4% (Ib) vs. 35%/2% (Chl) Ext. f/up: 92%/34% (Ib) vs. 37%/UK (Chl)	N/R	Median follow-up 82.7 months; median PFS NR (Ib) vs. 15 m (Chl), HR 0.154 (95% CI, 0.108–0.220) 7-year PFS rates: 59% (Ib) vs. 9% (Chl)	Median follow-up 82.7 months; median OS NR (Ib) vs 89 m (Chl), HR 0.453 (0.276–0.743)
ALLIANCE 202 [5, 53] Phase III, Patients ≥ 65y 1:1:1 Ib:IbR:BR Ib—until PD or intolerance, IbR: as per Ib, plus RTX C2-6, BR—intended six cycles	93%/7% (Ib) 94%/12% (IbR) 81%/26% (BR)	N/R	Median follow-up 55 months; median PFS 44 m (BR), NR (Ib), NR (IbR). IbR vs Ib, HR 0.99 (95% CI 0.66–1.48), (*p* = 0.96) 48-month PFS rates: 47% (BR), 76% (Ib), 76% (IbR)	48-month OS rates: 84% (BR), 85% (Ib), 86% (IbR)
ILLUMINATE [3, 35] Phase III, patients ≥ 65y or < 65 with pre-existing conditions IbO vs. ChlO, cross-over allowed upon PD IbO—Ib until PD or intolerance plus O C1-6, ChlO—intended six cycles	91%/42% (IbO) 81%/17% (ChlO)	Median follow-up 45 months; [PB or BM] 38% (IbO) vs. 25% (ChlO), (*p* = 0.033)	Median follow-up 45 months; median PFS NR (IbO) vs 22 m (ChlO), HR 0.25 (95% CI 0.16–0.38), (*p* < 0.0001) 42-month PFS rates: 74% (IbO) vs 33% (ChlO)	Median follow-up 45 months; NR vs NR, HR 1.08 (95% CI 0.60–1.97), (*p* = 0.793)
ELEVATE-TN [6, 57, 58] Phase III, patients ≥ 65y or < 65 with comorbidities (CrCl 30-69 ml/min or CIRS > 6) 1:1:1 AO:A:ChlO, A—until PD or intolerance, AO: as per A, plus O C1-6, ChlO—intended six cycles	Initial report: 94%/14% (AO), 86%/1% (A), 79%/5% (ChlO) 5-year f/up: 96%/32% (AO), 90/14% (A), 82%/14% (ChlO)	Patients achieving CR/CRi only; [PB] 49% (AO), 7% (A), 61% (ChlO) (lancet)	Median follow-up 58.2 months; median PFS NR (AO), NR (A), 27.8 m (ChlO). AO vs ChlO, HR 0.11, (*p* < 0.0001), A vs. ChlO HR 0.21, (*p* < 0.0001) 60-month PFS rates: 84% (AO), 72% (A), 21% (ChlO)	Median follow-up 58.2 months; median OS NR (all arms), AO vs ChlO, HR 0.55, (*p* = 0.0499) 60-month OS rates: 90% (AO), 84% (A), 82% (ChlO)
SEQUOIA [4, 129] Phase III, patients ≥ 65y or < 65 with comorbidities, without del(17p) 1:1 Z:BR, Z—until PD or intolerance, BR—intended six cycles	94.6%/6.6% (Z) vs 85.3%/15.1% (BR)—ash 2021	N/R	Median follow-up 26.2 months; NR vs. NR, HR 0.42 (95% CI 0.28–0.63), (*p* < 0.0001)	24-month OS rates: 94.3% (Z) vs. 94.6% (BR)
FLAIR [2] Phase III, patients < 75, no del(17p) > 20% cancer cell fraction 1:1 FCR:IbR (Parallel group trial), IbR—up to 6 years plus RTX × 6 (C1-6), FCR—intended six cycles	N/R	N/R	Median follow-up 52.7 months; median PFS NR (IbR) vs. 67 m (FCR), HR 0.44, (*p* < 0.001)	Median follow-up 52.7 months; no difference in median OS, HR 1.01, (*p* = 0.956)
E1912 [1, 26] Phase III, patients ≤ 70y without del(17p) 2:1 IbR:FCR, IbR—Ib until PD or intolerance, plus RTX × 6 (C1-6), FCR—intended six cycles	95.8%/17.2% (IbR) vs. 81.1%/30.3% (FCR)	12-month assessment; [PB] 8.3% (IbR) vs. 59.2% (FCR)	5-year PFS rates: 78% (IbR) vs. 51% (FCR), HR 0.37 (95% CI 0.27–0.51), (*p* < 0.0001)	5-year OS rates: 95% (IbR) vs. 89% (FCR), HR 0.47 (95% CI 0.25–0.89), (*p* = 0.018)
CLL13 [9, 10] Phase III, fit patients (CIRS ≤ 6, CrCl ≥ 70 ml/min) without *TP53* aberrancy 1:1:1:1 VenR:VenO:IbVenO:CIT VenR—12 months Ven plus RTX × 6 (C1-6), VenO—12 months Ven plus O × 8 (C1-6), IbVenO—as per VenO plus Ib C1-12, continued until cycle 36 if MRD +	Month 15: VenR: 93.3%/49.4% VenO: 96.1%/56.8% IbVenO: 94.4%/61.9% FCR: 80.8%/31.0%	Month 15: [PB] 57.0% (VenR), 86.5% (VenO), 92.2% (IbVenO), 52.0% (CIT) VenO vs CIT, *p* < 0.0001, IbVenO vs. CIT, *p* < 0.0001, VenR vs CIT, *p* = 0.317	Median observation time 38.8 months; IbVenO vs. CIT, HR 0.32, (97.5% CI 0.19–0.54), (*p* < 0.0001), VenO vs CIT HR 0.42, (97.5% CI 0.26–0.68), (*p* < 0.0001), VenR vs. CIT HR 0.79 (97.5% CI 0.53–1.18), (*p* = 0.183) Three-year PFS rates: 80.8% (VenR), 87.7% (VenO), 90.5% (IbVenO), 75.5% (CIT)	Median observation time 38.8 months; OS rates ‘similar across all treatment arms’
CLL14 [7, 8, 24] Phase III, patients with comorbidities (CIRS > 6 or CrCL < 70 ml/min) 1:1 VenO:ChlO, VenO and ChlO—12 cycles each, no cross-over allowed	84.7%/49.5% (VenO) vs. 71.3/21.3% (ChlO)	Month 15^#^: [PB] 75.5% (VenO) vs. 35.2% (ChlO), (*p* < 0.001) [BM] 56.9% (VenO) vs. 17.1% (ChlO), (*p* < 0.001)	Median follow-up 65.4 months; median PFS NR vs 36.4 months, HR 0.35 (95% CI 0.26–0.46), (*p* < 0.0001) 5-years PFS rates: 62.6% (VenO) vs. 27.0% (ChlO)	5-year OS rates: 81.9% (VenO) vs. 77.0% (ChlO), HR 0.72 (95% CI 0.48–1.09), (*p* = 0.12)
CAPTIVATE [52, 103] Phase II, patients 18-70y Intended 3 cycles Ib, 12 cycles of IbVen *FD cohort:* If PD < 24 months—could receive Ib, if PD > 24 months—could be retreated with IbVen	96%/55%	EOT: [PB] 77% [BM] 60%	24-month PFS rates: 95% (all-treated), 96% (without del[17p]), 84% (with *TP53* aberrancy)	24-month OS rates: 98% (all-treated), 98% (without del[17p]), 96% (with *TP53* aberrancy)
*MRD-cohort*: One additional cycle of IbVen—MRD status confirmed and response assessed; uMRD Confirmed: 1:1 placebo:Ib until confirmed MRD relapse uMRD Not Confirmed: 1:1 to Ib:IbVen (maximum 2 years overall duration for venetoclax) until PD or intolerance	97%/46%	EOT: [PB] 75% [BM] 68%I	30-month PFS rates: ‘uMRD confirmed’ = 95% (placebo) vs. 100% (Ib) ‘uMRD Not Confirmed’—95% (Ib) vs. 97% (IbVen)	N/R
GLOW [32, 34] Phase III, patients ≥ 65y or < 65y with CIRS ≥ 6 or CrCl < 70 ml/min, without known TP53 aberrancy 1:1 IbVen:ChlO, IbVen—intended 12 cycles after three cycles Ib lead-in, ChlO—intended six cycles	86.8%/38.7% (IbVen) vs. 84.8%/11.4% (ChlO) (IRC)	[BM] 55.7% (IbVen) vs. 21.0% (ChlO)^#^	Median follow-up 27.7 months; median PFS IbVen vs ChlO, HR 0.216 (95% CI 0.131–0.357), (*p* < 0.001) 24-month PFS rates: 84.4% (IbVen) vs. 44.1% (ChlO)	Median follow-up 27.7 months; median OS—‘no difference in overall survival between arms’, HR 1.048 (95% CI 0.454–2.419)

*UK* unknown, *NR* not reached, *N/R* not reported, *FD* fixed duration, *ORR/CR+Cri* overall response rate/complete response plus complete responses with incomplete haematological recovery, *PFS* progression-free survival, *OS* overall survival, *MRD* measurable residual disease, *O* obinutuzumab, *R* rituximab, *Chl* chlorambucil. Ibrutinib (Ib) administered at 420 mg once daily ongoing, acalabrutinib (A) administered at 100 mg twice daily ongoing, and zanubrutinib (Z) 160 mg twice daily ongoing unless otherwise stated. Venetoclax (Ven) administered at 400 mg OD after dose ramp-up unless otherwise indicated. MRD measured by multiparametric flow cytometry (threshold < 10^−4^) unless otherwise indicated by ^#^ where assessed by next-generation sequencing

However, outcomes for patients with high-risk genomic lesions, especially those with *TP53* aberrancy, are well recognized to be inferior following CIT irrespective of regimen intensity or patient fitness. The presence of del(17p) and/or *TP53* mutations were strongly associated with inferior PFS (median PFS of 11.2 months) following FCR in CLL8 [18]. As such, patients with *TP53* aberrant CLL were appropriately excluded from the CLL10 study. Patients with unmutated IGHV (umIGHV) CLL compared with IGHV-mutated disease demonstrated inferior PFS following FCR (CLL8, 5-year PFS rates of 33.1% vs. 66.6%) [18, 22], BR (CLL10, mPFS 33.9 vs. 68.9 months, HR 2.431 (95% CI 1.674–3.530), *p* < 0.0001) [19, 21], and chlorambucil (Chl) regimens (CLL11, HR 1.97 (95% CI 1.52–2.55), *p* < 0.001) [23]. While the adverse prognostic impact of *TP53* aberrancy and umIGHV are also retained following VenO as first therapy [24], VenO achieves superior PFS compared with CIT for both subgroups in CLL14 [7] and CLL13/GAIA [9, 25]. Similarly, patients with umIGHV CLL treated with ibrutinib-rituximab (IbR) in E1912 [1, 26] and FLAIR studies [27] observed improved PFS compared with CIT. While no studies comparing BTKi and CIT have included significant numbers of patients with *TP53* aberrant disease, pooled PFS outcomes of such patients treated with ibrutinib (Ib) are superior to those reported in CLL8 following FCR [28].

However, although representing the minority, FCR may still be justifiably offered as an alternative to novel therapies for young, fit patients with mIGHV CLL and absence of *TP53* aberrancy with the aim of achieving long-term disease control. Most may be successfully transitioned to novel therapies following relapse [29, 30]. In a Phase II study of FCR, although no differences were observed in CR rates, patients with mIGHV CLL achieved superior rates of bone marrow (BM) undetectable measurable residual disease (uMRD) by polymerase chain reaction (PCR) compared with umIGHV, 50.7% versus 33% [31]. Long-term follow-up of the mIGHV group demonstrated sustained PFS of 53.9% at 12.8 years; no relapses observed in 42 patients beyond 10.4 years follow-up with plateaued Kaplan–Meier estimates. Although no association between IGHV mutational status and rates of complete response, uMRD, or OS were observed in CLL8, significant difference in PFS following FCR was demonstrated in favor of mIGHV compared with umIGHV, with apparent plateau in PFS estimate (median PFS not reached) after 7 years of follow-up [18]. These data provoke the question as to whether FCR as first therapy may cure a significant portion of patients with mIGHV CLL.

Whether long-term PFS outcomes for mIGHV CLL may be similar following FCR or novel therapies is uncertain. The E1912 and FLAIR studies demonstrated discordant PFS outcomes for mIGHV patients when comparing Ib (± rituximab [Ritux]) to FCR [9, 27]. In E1912, 5-year PFS for mIGHV patients favored Ib (83% vs 68%; HR = 0.27, *p* = 0.001); however, no difference was observed in FLAIR (HR 0.64 [0.35–1.16], *p* = 0.15) after median 52.7-month follow-up. It is important to highlight the shorter follow-up periods for these studies at which point any potential plateau in Kaplan–Meier PFS estimates for mIGHV patients were yet to be observed in the MD Anderson cohort and CLL8. PFS outcomes by IGHV mutational status following IbVen have not yet been reported by either GLOW or FLAIR studies; however, patients with umIGHV CLL appear to achieve superior rates of uMRD [32, 33].

Novel therapies consistently demonstrate superior PFS compared with less intensive CIT (obinutuzumab-chlorambucil [ChlO]/BR) for unselected unfit/older patients [4, 7, 34, 35]. However, older patients may express differing goals of therapy including prioritization of time-limited treatment exposure. Where BTKi and/or VenO are not readily available, ChlO may still be offered with reasonable efficacy for patients without *TP53* aberrant CLL, particularly for the minority of patients with mIGHV CLL. After median 42.4-month follow-up, median time to next treatment (TTNT) was 51.1 months following six cycles of ChlO for all older patients in CLL11 [36]. Meaningful PFS (median not reached) was achieved for elderly/comorbid patients with mIGHV CLL treated with either ChlO or ibrutinib-obinutuzumab (IbO) in iLLUMINATE after 45-month follow-up [3]. A retrospective analysis of patients treated with Ib or ChlO demonstrated significant PFS and TTNT differences favoring Ib after propensity matching; however, no difference in PFS or TTNT was observed between the two therapies for mIGHV patients [37]. However, higher rates of hematological toxicity have been observed with ChlO as compared with Ib or VenO in older patients [3, 7].

When selecting CIT, particularly FCR, as initial therapy, it is paramount to counsel the patient about potential short- and long-term toxicities. Discussion about risks must include high rates of hematological toxicity and infection, but must importantly extend to increased risk of secondary myeloid malignancies [18]. Incidence of myeloid malignancies was comparatively higher in long-term follow-up of CLL10 for FCR compared with BR (2.2% vs. 0.4%). Furthermore, CIT (particularly fludarabine-containing) is associated with adverse lymphoid clonal evolution, e.g., complex karyotype, CLL-associated mutations compared with VenO [38], and hence may conceptually increase future risk of Richter transformation [39]. The risks must also be balanced against those risks associated novel therapies including the incidence of non-hematological malignancies, fatal and non-fatal arrhythmias with Ib, cumulative hypertension with BTKi, and tumor lysis syndrome with Ven. Notwithstanding, these risks may be deemed acceptable given the possibility of durable remission with time-limited therapy which may appeal to younger, active patients. Given significant costs associated with novel therapies which may result in out-of-pocket charges for patients in some jurisdictions [40], irrespective of traditional toxicities CIT may retain also relative appeal when financial toxicity is considered.

## When using a BTKi, which is the preferred agent?

Bruton’s tyrosine kinase is important for normal B-cell receptor signaling culminating in upregulation of pathways supporting B-cell survival and proliferation [41, 42]. The covalent BTKi Ib which binds irreversibly to the C481 residue of the ATP-binding domain of BTK was the first approved BTKi. Following high overall response rates and superiority to ofatumumab in the relapsed/refractory setting [43, 44], Ib was associated with significantly superior PFS and OS compared with Chl as first therapy for CLL in the phase III study RESONATE-2, establishing it as a standard of care first therapy [45]. Significant PFS differences favoring Ib have been retained in more recent studies as compared with CIT combinations regardless of patient fitness, namely ChlO in iLLUMINATE [3], BR in ALLIANCE 202 [5], and FCR in E1912 [26] and FLAIR studies [2]. Of these studies, only E1912 has demonstrated OS benefit favoring Ib; however, lack of apparent benefit in the other studies may be attributed to study cross-over design and/or improved subsequent lines of therapy compared with historical norms.

Ib and other BTKis are administered as continuous therapy until disease progression or intolerance. High rates of treatment discontinuation are observed with long-term Ib follow-up, see Table 1 [45]. Rates of discontinuation are significantly higher in real-world series—65.2% of patients treated with Ib (*n* = 11,870) had discontinued therapy after median 25.2 months [46] and estimated 41% of 546 patients had discontinued therapy after median 17 months in a second large cohort [47]. The majority of discontinuations were early and attributed to adverse events/intolerance in both studies. Increased incidence of adverse events such as bleeding, diarrhea, rash, and arthralgias are directly attributable to inhibition of specific off-target kinases, e.g., EGFR, TEC [48]. Cardiovascular complications are important BTKi-related adverse events, including hypertension, atrial fibrillation (AF)/flutter, and importantly both ventricular arrhythmias and risk of sudden death, see Table 2. Most afflicted patients have baseline risk factors or AF, pre-existing hypertension, or related disorders [44, 49]. Significantly increased (approximately eightfold compared with age-matched population) [50] but low absolute incidence (approximately 1/100–300) [49, 51] of sudden death, presumed largely from ventricular arrhythmia, are common features of studies using Ib [2, 34, 44, 45, 52, 53]. Although one retrospective single-center study suggested that acalabrutinib is similarly associated with increased incidence of ventricular arrythmias compared with the general population, the significance of the arrythmias (largely PVCs) described is unclear and no clear association with sudden cardiac death was established from these data [54].

**Table 2:** Important safety outcomes of pivotal clinical trials in untreated CLL

Pivotal studies	All any-grade/grade ≥ 3 adverse events	Any-grade/grade ≥ 3 neutropenia; any-grade/grade ≥ 3 infection	Any-grade/grade ≥ 3 thrombocytopenia; any-grade/grade ≥ 3 bleeding	Any-grade/grade ≥ 3 diarrhoea	Any-grade/grade ≥ 3 atrial fibrillation	Any-grade/grade ≥ 3 hypertension	Treatment discontinuation due to AE Treatment related deaths
CLL8 [18, 20]	UK/63% (FC) vs. UK/73% (FCR))	Neutropenia: UK/21% (FC) vs. UK/34% (FCR) Infection: UK/21% (FC) vs. UK/25% (FCR)	Thrombocytopenia: UK/11% (FC) vs. UK/7% (FCR) Bleeding: N/R	N/R	N/R	N/R	Discontinuation: UK/17% overall Death: 3% (FC) vs. 2% (FCR)
CLL10 [18]	92%/UK (BR) vs. 98%/UK (FCR)	Neutropenia: 60%/59% (BR) vs. 76%/75%% (FCR) Infection: 67%/26% (BR) vs. 77%/40% (FCR)	Thrombocytopenia:18%/14% (BR) vs. 24%/21% (FCR) Bleeding: N/R	N/R	N/R	N/R	Discontinuation: 13% (BR) vs. 23% (FCR) Death: 2% (BR) vs. 5% (FCR
RESONATE-2 [45, 128]	N/R	Neutropenia: 16%/10% (Ib) vs. 23%/18% (Chl) Infection: N/R	Thrombocytopenia: UK/2% (Ib) vs. UK/2% (Ib) Bleeding: UK/4% (Ib) vs. UK/2% (Chl) Ext. f/up UK/7% (Ib)	42%/4% (Ib) vs. 17%/0% (Chl)	6%/1.5% (Ib) vs. 1%/UK (Chl) Ext. f/up—UK/6% (Ib)	14%/4% (Ib) vs. UK/0% (Chl) Ext. f/up—UK/12% (Ib)	Discontinuation: 9% (Ib) vs. 23% (Chl) Ext. f/up—24% (Ib) Death: N/R 3% fatal cardiac events (Ib)
ALLIANCE 202 [53]	Haematologic: UK/41% (Ib), UK/39% (IbR), UK/61% (BR) Non-haematologic: UK/74% (Ib), UK/74% (Ib), UK/63% (BR)	Neutropenia: UK/15% (Ib), UK/21% (IbR), UK/40% (BR) Infection: UK/18% (Ib), UK/20% (IbR), UK/15% (BR)	Thrombocytopenia: UK/7% (Ib), UK/5% (IbR), UK/15% (BR) Bleeding: UK/2% (Ib), UK/4% (IbR), UK/0% (BR)	N/R	17%/9% (Ib), 14%/6% (IbR), 3%/3% (BR)	UK/29% (Ib), UK/33% (IbR), UK/15% (BR)	Discontinuation: N/R Death: Sudden deaths 4% (Ib), 2% (IbR), 1% (BR)
ILLUMINATE [3, 35]	90%/68% (IbO) vs. 95%/70% (ChlO)	Neutropenia: 44%/37% (IbO) vs 63%/46% (ChlO) Infection: N/R	Thrombocytopenia: 35%/19% (IbO) vs. 25%/10% (ChlO) Bleeding: IbO: 4%/UK (IbO) vs. UK/UK (ChlO)	34%/3% (IbO) vs 10%/0% (ChlO) Ext. f/up—35%/3% (IbO)	7%/5% (IbO) vs. 0%/0% (ChlO) Ext. f/up—15%/6% (IbO)	13%/4% (IbO) vs. 4%/3% (ChlO) Ext. f/up—19%/4% (IbO)	Discontinuation: 16% (IbO) vs. 9% (ChlO) Ext. f/up—22% (IbO) Death: 12.4% (IbO) vs. 2.6% (ChlO)
ELEVATE-TN [6, 57, 58]	96.1%/70.2% (AO), 95.0%/49.7% (A), 98.8%/69.8% (ChlO)	Neutropenia: 31.5%/29.8% (AO), 10.6%/9.5% (A), 45%/41.4% (ChlO) Infection: UK/21% (AO), UK/14% (A), UK/8% (ChlO)	Thrombocytopenia: 12.9%/8.4% (AO), 7.3%/2.8% (A), 14.2%/11.8% (ChlO) Bleeding: 43%/2% (AO), 39%/2% (A), 12%/0% (ChlO)	38.8%/4.5% (AO), 34.6%/0.6% (A), 21.3%/1.8% (ChlO)	3%/UK (AO), 4%/UK (A), 1%/UK (ChlO	UK/3% (AO), UK/2% (A), UK/3% (ChlO)	Discontinuation: 11% (AO), 9% (A), 14% (ChlO) Ext. f/up: 17% (AO), 16% (A), 14% (ChlO) Death: 2.2% (AO), 3.6% (A), 6.5% (ChlO)
SEQUOIA [4]	94%/53% (Z) vs. 96%/80% (BR)	Neutropenia: 16%/12% (Z) vs 57%/51% (BR) Infection: UK/16.3% (Z) vs. UK/18.9% (BR)	Thrombocytopenia: 3–4%/1–2% (Z) vs. 13%/7% (BR) Bleeding: 45%/4% (Z) vs. 11%/1.8% (BR)	14%/1% (Z) vs. 13%/1% (BR)	3.3%/0.4% (Z) vs. 2.6%/1.3% (BR)	14.2%/6.3% (Z) vs. 10.6%/4.8% (BR)	Discontinuation: 8% (Z) vs. 14% (BR) Death: 4.6% (Z) vs.5.3% (BR)
FLAIR [2]	N/R	Neutropenia: N/R Infection: 27.1%/UK (FCR) vs 33.6%/UK (IbR)	N/R	N/R	All cardiac adverse events; 1.1% (FCR) vs. 8.3% (IbR)	Discontinuation: N/R Death: Sudden deaths—2.1% (IbR) vs. 0.5% (FCR)	
E1912 [1, 26]	UK/80.1% (IbR) vs. UK/79.7% (FCR)	Neutropenia: UK/25.6% (IbR) vs. UK/44.9% (FCR) Ext. f/up—UK/28.4% (IbR) Infection: UK/9.1% (IbR) vs. UK/8.9% (FCR) Ext. f/up—UK/11.4% (IbR)	Thrombocytopenia: UK/3.3% (IbR) vs. UK/36.8% (FCR) Bleeding: UK/1.1% (IbR) vs. UK/0% (FCR)	UK/4.3% (IbR) vs. UK/1.3% (FCR)	7.4%/UK (IbR) vs. 3.2%/UK (FCR) Ext. f/up—UK/4.5% (IbR) vs. UK/0% (FCR)	UK/18.8% (IbR) vs. UK/ 8.2% (FCR)	Discontinuation: N/R Ext. f/up—21.9% (IbR) (2022) Death: 1 sudden death in IbR arm
CLL13 [10]	96.6%/71.3% (VenR), 98.7%/84.7% (VenO), 98.7%/82.2% (IbVenO), 98.1%/78.7% (CIT)	Neutropenia: 53.2%/46.0% (VenR), 58.8%/55.7% (VenO), 56.7%/48.5% (IbVenO), 55.6%/52.3% (CIT) Infection: 59.5%/11.4% (VenR), 68%/14% (VenO), 75.3%/22.1% (IbVenO), 60.6%/19.9% (CIT)	Thrombocytopenia: 10.1%/4.2% (VenR), 23.2%/18.4% (VenO), 29.9%/16.0% (IbVenO), 19.0%/10.2% (CIT) Bleeding: 5.1%/0.4% (VenR), 10.1%/0.4% (VenO), 27.7%/1.7% (IbVenO), 6.0%/0.5% (CIT)	N/R	0.8%/0.4 (VenR), 0.9%/0.0% (VenO), 7.8%/2.6% (IbVenO), 1.9%/0.5% (CIT)	N/R	Discontinuation: N/R Death: N/R
CLL14 [7]	VenO: UK/78.8% ChlO: UK/76.6%	Neutropenia: UK/52.8% (VenO) vs UK/48.1% (ChlO) Infection: UK/17.5% (VenO) vs. UK/15.0% (ChlO)	Thrombocytopenia UK/13.7% (VenO) vs. UK/15.0% (ChlO) Bleeding: N/R	UK/4.2% (VenO) vs. UK/0.5% (ChlO)	N/R	N/R	Discontinuation: N/R Death: N/R
CAPTIVATE (FD cohort) [52]	N/R	Neutropenia: 42%/33% Infection: 67%/8%	Thrombocytopenia: 59%/13% Major bleeding: 2%/1%	62%/3%	4%/1%	16%/6%	Discontinuation: N/R Death: N/R
GLOW [34]	UK/75.5% vs. UK/69.5%	Neutropenia: 41.5%/34.9% (IbVen) vs. 58.1%/49.5% (ChlO) Infection: UK/17.0% (IbVen) vs. UK/11.5% (ChlO)	Thrombocytopenia: 11.3%/5.7% (IbVen) vs. 26.7%/20.0% (ChlO) Bleeding: N/R	50.9%/10.4% (IbVen) vs. 12.4%/1.0% (ChlO)	14.2%/6.6% (IbVen) vs. 1.9%/0.0% (ChlO)	13.2%/7.5% (IbVen) vs. 4.8%/1.9% (ChlO)	Discontinuation: N/R Death: 6.6% (IbVen) vs. 1.9% (ChlO)

Same abbreviations used as in Table 1

Next-generation covalent BTKi Acala and zanubrutinib (Zanu) also demonstrate superiority with respect to PFS compared with CIT. Both were developed to act with greater selectivity and less off-target kinase inhibition with the aim of reducing associated toxicity burden. Both agents exhibit stable and near-complete BTK occupancy with recommended dosing [55, 56]. The 5-year follow-up of ELEVATE-TN confirmed a significant PFS benefit favoring AO or A over ChlO and OS advantage for AO over ChlO, see Table 1 [57]. With shorter follow-up, the SEQUOIA study demonstrated superior PFS (24-month rate 86% vs. 70%, *p* < 0.0001) but not OS following Zanu compared with BR in treatment-naïve patients with CLL without del(17p) [4]. While rates of treatment discontinuation with next-generation BTKi appear improved compared with Ib, they remain significant in 1L studies, see Table 1 [4, 58].

Head-to-head comparisons of Acala versus Ib (ELEVATE RR) [59] and Zanu versus Ib (ALPINE) [60] in R/R CLL, and Zanu versus Ib for patients with Waldenstrom macroglobulinaemia (ASPEN) [61], give insights into comparative efficacy and toxicity profiles of approved BTKi. Acalabrutinib demonstrated non-inferior PFS to Ib after median follow-up of 40.9 months (HR 1.00 [95% CI 0.79–1.27]) [59], however with comparatively lower rates of treatment discontinuation (14.7% vs. 21.3%) and lower frequencies of common adverse events and cardiac events overall, see Tables 1 and 2. This included a 48% lower cumulative incidence of atrial fibrillation/flutter, and lower cumulative incidence of diarrhea and arthralgias, and fewer hypertension events [59]. A post-hoc analysis of these data evaluated the burden of adverse events by incorporating the duration and weighted severity of events observed confirmed the differences reported [62].

In ALPINE, overall response rates were higher with Zanu versus Ib, including in patients with *TP53* aberrant CLL (80.5% vs. 50.0%), see Table 1 [63]. Investigator-assessed PFS favored Zanu after median follow-up of 29.6 months; [60] superiority in PFS was sustained in all major patient subgroups (according to age, previous lines of therapy, stage, IGHV mutational status) including those with *TP53* aberrancy, HR 0.53 (95% CI 0.31–0.88). Overall, events leading to treatment discontinuation and numbers of cardiac events leading to treatment discontinuation or death were lower with Zanu, see Table 2. Incidence of any grade atrial fibrillation/flutter was lower with Zanu (5.2% vs 13.3%) [60], consistent with observations in ASPEN [61]. Six deaths due to cardiac events were reported in the Ib group (none with Zanu), all of whom had pre-existing cardiac issues, four of which occurred within four months of initiating Ib. No difference in hypertension of any grade including grade ≥ 3 events was observed (Table 2) [60], in contrast to the twofold increase in all hypertension events demonstrated with Ib compared with Zanu (*p* = 0.16) following exposure adjustment in ASPEN [61].

Overall, while there are data supporting efficacy all three covalent BTKi in the treatment-naïve setting, Zanu and Acala are the preferred BTKi due to at least similar efficacy and reduction in important toxicities compared with Ib in the R/R setting. There are specific situations in which these preferences may be stronger such as those with pre-existing cardiac comorbidities and/or those receiving concomitant anticoagulant or antiplatelet therapy. Fewer any-grade cardiac events resulting in treatment discontinuation or death were observed with both Acala and Zanu compared with Ib. Ib should be avoided with use of Vitamin K antagonists such as warfarin due to interference with CYP3A4 metabolism and the fatal hemorrhages reported for warfarin-treated patients in early Ib trials [43, 64]. Fewer bleeding events, although similar frequency of major bleeding, were observed with Acala versus Ib in ELEVATE-RR [59]. While incompletely understood, it is postulated that the reduction in cardiovascular adverse events derives from reduced SRC kinase inhibition with Zanu [56, 65].

It is important to recognize the ‘dead-kinase’ mutations at codon L528 of *BTK* which have been observed with disease progression on Zanu and pirtobrutinib (Pirto) [66–68]. These have been described at lower frequency with Ib or Acala. They may have important implications for double class-refractory disease for whom effective treatment options are limited. One emerging class of agents for double-refractory is the non-covalent BTKi (e.g., Pirto), which demonstrate efficacy in covalent-BTKi-resistant CLL harboring secondary C481 *BTK* mutations [69–71]. Dead-kinase L528 mutations following Zanu may induce cross-resistance to Pirto [66]. However, the precise incidence of L528 *BTK* mutations arising following Zanu therapy is presently undefined and should not currently influence selection of initial BTKi until more data are available.

## When using a BTKi, should you add an anti-CD20 monoclonal antibody?

While Ven-anti-CD20 monoclonal antibody (mAb) combinations are established standards of care for both TN and R/R CLL, less is known about the merits of adding an anti-CD20 monoclonal antibody to BTKi as first therapy.

The addition of Ritux and Obi to Ven has facilitated fixed-duration Ven therapy, evolving from continuous Ven therapy first evaluated in R/R CLL [72, 73]. Achieving uMRD with Ven therapy is associated with superior duration of response and PFS. The addition of Ritux to Ven appears to augment rates of complete response and uMRD achieved [30, 72, 74–76]. In a phase 1b study of VenR, 5-year estimates of ongoing response for deep responders (CR/CRi or uMRD) were similar following continuous or fixed-duration Ven (median treatment duration 1.4 years) after six cycles of VenR [77]. For TN and R/R CLL, fixed-duration VenO and VenR demonstrated superior PFS to CIT in the CLL14 and MURANO studies [7, 30].

Whether the addition of anti-CD20 monoclonal antibody to BTKi may deepen responses to allow for time-limited therapy [78] or augment duration of responses observed with continuous therapy are uncertain. In contrast to Ven, uMRD rates are low following BTKi and achieving uMRD is not associated with superior duration of response or PFS [79]. A phase II study of Ib demonstrated 4-year PB uMRD rates of 10.2%, with 57% of patients remaining on treatment [80]. Although median BM MRD was lower in another phase II study for patients treated with IbR versus Ib (19.8% vs 12.2%, *p* = 0.0180), no significant difference in rates of CR (*p* = 0.32) nor difference in 36-month PFS rates (86.9% vs. 86%, *p* = 0.912) were observed [81].

Combinations of BTKi-R/O have been recently evaluated in several 1L clinical studies in both fit and unfit patients with CLL. The iLLUMINATE and E1912 studies demonstrate that IbO and IbR, respectively, are superior to CIT with respect to PFS. While uMRD rates remained low with IbR in E1912, higher rates were observed with IbO in iLLUMINATE see Table 1 [3]. However, in both studies, achieving uMRD did not associate clearly with improved PFS outcomes [1, 3]. Continuous Ib monotherapy was compared with IbR (continuous Ib with six cycles of Ritux) in ALLIANCE 202 (A41702); after median follow-up of 55 months, PFS was identical between the two arms (HR 0.99, *p* = 0.96) [5]. The lack of benefit with the addition of Ritux was observed in all high-risk subgroups examined including those with *TP53* abnormalities [5].

In ELEVATE-TN, the addition of Obi to Acala (AO) appeared to improve rates of CR/CRi at 4-year follow-up (AO [30.7%] vs. A [11.2%]), including CR/CRi rates of 32.0% vs. 13.0% for *TP53* aberrant CLL and 28.2% versus 12.6% for umIGHV [6]. In an underpowered *post-hoc* analysis, prolonged PFS was observed with AO versus A (*p* = 0.0296) including greater 48-month PFS benefit when individually compared with PFS following ChlO (HR 0.1 vs HR 0.19). Although not compared directly, superior PFS rates were sustained at 60-month follow-up (AO [84%] vs. A [72%]) [57]. However, no specific subgroup of patients was observed to have statistically significant benefit with AO compared with A monotherapy [6].

While there are no compelling data to suggest the addition of Ritux to Ib improves efficacy, AO may prove superior to Acala monotherapy. There are no available randomized data to inform the combination of IbO vs Ib, and there are limitations to cross-comparison data comparison due to differences in monitoring and assessment between studies published. There is preclinical premise for the differential uMRD rates and PFS benefit seen between the two BTKi-antiCD20 combinations. Rituximab exhibits anti-CLL activity through complement-dependent cytotoxicity (CDC), antibody-dependent cellular cytotoxicity (ADCC), and direct induction of programmed cell death (PCD) [82]. By comparison, Obi induces greater potent ADCC and PCD, but lesser CDC [83]. Ib may impair rituximab-induced ADCC due to inhibition of ITK, which may explain lack of PFS benefit with the combination. However, Obi may provide added benefit to Acala due to baseline superior ADCC potency and/or reduced ITK inhibition through improved on-target specificity. Obinutuzumab was associated with greater cell death compared with Ritux in MCL cell lines co-cultured with Ib [84].

However, the potential benefits of AO versus A currently appear marginal and must be balanced against the increase in adverse effects observed with the combination, the inability to identify any subgroups with greater likelihood of benefit, as well as the added logistic burdens of the IV infusions. Although no difference in treatment continuation due to adverse events was observed at 4-year follow-up, more frequent grade ≥ 3 adverse events were observed with AO vs A at primary analysis [6, 58]. Greater incidence of hematological adverse events was observed following AO versus A, including all-grade neutropenia and thrombocytopenia. Incidence of grade ≥ 3 infection and contusion, but not grade ≥ 3 bleeding events was increased following AO versus A, see Table 2. Of those who received AO, 13.5% experienced and infusion-related reaction [58].

We suggest that on current evidence Ritux should not be routinely added to BTKi monotherapy. Although Obi has a possible role in combination with Acala, the lack of demonstrable PFS benefit for key subgroups raises uncertainty as to whether the greater potential for hematological and infectious adverse events can be justified.

## Is continuous BTKi or time-limited Ven-based therapy preferred?

Both BTKi and VenO are associated with superior PFS when compared with CIT as first therapy for patients with CLL; the observed PFS benefit is sustained for all high-risk genetic subgroups for both therapies. For patients with mIGHV CLL, similar PFS is demonstrated between VenR (but not VenO) and CIT (FCR/BR) [9], and there is discordance between possible PFS benefit seen following IbO compared with FCR between the E1912 and FLAIR studies [1, 2]. The presence of *TP53* aberrancy [24] and umIGHV [9, 24] is associated with inferior PFS following VenO. The presence of high-risk genetic features does not clearly influence survival outcomes following BTKi [3, 5, 6]. However, to date there are no published 1L head-to-head studies which have compared the efficacy or safety of these agents directly in unselected patients or any high-risk subgroups. No data are yet available from CLL17 study (NCT04608318) which importantly seeks to compare efficacy (primary endpoint PFS) of three regimens; Ib continuous monotherapy vs fixed-duration VenO vs fixed-duration IbVen.

While perceived efficacy of therapy is a key driver of therapeutic decisions for CLL, more immediate patient-specific factors may be equally, if not more, important when selecting between novel first therapies. It is important to consider general fitness and specific comorbidities, adverse event profiles described, and perceived tolerability, acknowledging discontinuation rates due to adverse events in pivotal studies. Further factors to consider include the logistics of treatment administration, frequency of safety monitoring by venipuncture and/or clinic attendances, and potential burden placed on the patient and their carers. More broadly, it is also important to appreciate the ability to bidirectionally sequence novel therapies (i.e., Ven → BTK or BTK → Ven), appreciating the longitudinal context of the patient’s age and fitness.

Ven-antiCD20 combinations are appealing due to time-limited administration, high rates of uMRD predictive of durable remissions, and acceptable toxicity profile in older/unfit patients [7, 9, 30]. Time-limited therapy may be desirable due to perception of more transient impact on life commitments. The more immediate logistical burden of Ven ramp-up is well-established with respect to rigorous therapeutic monitoring including the potential for inpatient admission, and prophylactic strategies to mitigate risk of tumor lysis syndrome (TLS), particularly for those patients with higher tumor burden. Ability to receive Ven may therefore be restricted by patient transport or social factors, or resource limitations of the treating healthcare institution. While the frequency of TLS events is low in recent phase III studies of VenO and VenR [7, 9, 30], real-world data report greater incidence in the context of variable adherence to recommended TLS prophylactic measures [47, 85, 86]. However, beyond completion of ramp-up and attendance for intravenous anti-CD20 monoclonal antibody, the intensity of monitoring throughout remaining treatment is relatively unintrusive. Neutropenia may be successfully managed with dose interruptions, intermittent use of colony stimulating factor and eventually dose reduction if required, and reported rates of grade ≥ 3 febrile neutropenia are low following VenO (CLL14 5.2%; CLL13/GAIA 3.1%).

There are recent data and current studies evaluating the potential for Ven re-treatment after previous time-limited Ven-combination therapy. If selecting VenO as first therapy, ‘time to continuous therapy’ with subsequent BTKi may potentially be extended by this approach. Small numbers of patients treated with VenR in a phase 1b study and MURANO were re-treated with uMRD remissions achieved for the second time [77, 87]. An ORR of 79.5% was observed following second Ven therapy in a retrospective series of 39 evaluable patients compared with 95.7% ORR to the first Ven treatment [88]. Acquisition of resistance mutations impacting the binding groove of the BCL2 protein, e.g., Gly101Val and others are an established mechanism of secondary resistance to continuous Ven therapy [89, 90], however are not presently described following 12–24 months of fixed-duration therapy. These data beg the question whether reported time to next treatment following time-limited Ven-combinations should encompass clinical benefit derived following attempt at retreatment in the future.

In comparison, logistics of BTKi initiation are agnostic to pre-treatment disease burden and do not require up-titration of dosing. However, the continuous BTKi treatment paradigm is associated with significant cost, continual exposure to potential toxicities, and ongoing selection pressure likely impacting disease clonal evolution. The economic burden of BTKi for CLL may be especially high for younger patients who may be at reduced risk of all-cause mortality and eventually discontinue BTKi due to disease progression rather than adverse event. This cost burden is likely not restricted to healthcare systems and likely creates cumulative out-of-pocket cost to patients themselves [40].

The comparatively longer follow-up of pivotal trials of continuous BTKi demonstrates their cumulative toxicities and high rates of treatment discontinuation as previously discussed in “When using a BTKi, which is the preferred agent?” section. For example, 30–40% of patients will experience diarrhea with Ib or Acala [6, 45]. Although potentially tolerable during fixed-duration therapy, any burden of ongoing toxicity without a clear end-point is understandably challenging for patients. The drug-class associated adverse events supraventricular arrhythmias and hypertension are associated with increased mortality [91]. Patients with risk factors of older age, male sex, pre-existing hypertension, and valvular heart disease are at higher baseline risk of developing atrial arrhythmias [92]. Additionally, those with structurally abnormal hearts, e.g., following myocardial infarction, with cardiomyopathy or cardiac hypertrophy, congenital heart abnormalities, or with valvular disease are at heightened risk of ventricular tachyarrhythmias [93]. Concomitant use of dual antiplatelet therapy, anticoagulants, or pre-existing history of hemorrhage/bleeding diathesis should be carefully reviewed. The presence of significant cardiac comorbidities requires specific patient risk counselling and should prompt consideration of alternative first therapies for CLL.

The continuous BTKi treatment paradigm provides ongoing selection pressure from which clonal diversity arises. Growth of clones harboring resistance mechanisms such mutations within the genes coding the target protein (e.g., C481 *BTK* variants) or downstream pathway proteins, e.g., *PLCG2* herald treatment failure. Marked clonal shifts were noted to occur in nearly one-third of high-risk CLL treated with Ib; greater chance of secondary resistance mutations acquisition through greater clonal diversity is thought to be associated with *TP53* mutation-related genomic instability [94, 95].

For these reasons, we generally support the use of time-limited VenO over BTKi monotherapy as first therapy, particularly for younger patients. Comparable efficacy with lower cost, lower potential for adverse events, and the potential for retreatment are compelling reasons for selection in most cases.

## If pursuing time-limited therapy, is VenO or Ven-BTKi preferred?

The combination of Ven with either anti-CD20 mAb or BTKi affords deeper responses and delivery of fixed-duration treatment. While anti-CD20 monoclonal antibodies have relatively modest efficacy against CLL [82] as monotherapy as compared with other lymphoid malignancies like follicular lymphoma, VenO or VenR are highly effective combinations as discussed in previous sections. The combination of the two most effective classes of therapy available (BCL2i and BTKi), or even triplet therapy as discussed in "Is BTKi-BCL2i-antiCD20 triplet preferable to VenO?" section, seek to achieve superior depth and durability of responses seen with BTKi monotherapy or Ven-antiCD20 alone.

BTKi and BCL2i exhibit marked synergism in pre-clinical CLL models [96, 97], in addition to exhibiting independent mechanisms of anti-CLL activity. BTKi increase CLL dependence on BCL2 by downregulating the other key anti-apoptotic proteins MCL1 and BCL-X_L_ which are known to contribute to primary sensitivity to Ven and secondary resistance [98]. Potential efficacy with fixed-duration Ven-BTKi must, however, be balanced against the potential for augmented adverse events and the, at least theoretical, risk of resistance against one or both classes of therapy. The efficacy of BCL2i or BTKi as second novel therapy after initial use of the alternate class is well described [30, 99, 100]; however, there are uncertainties about the ability to salvage disease exposed to both classes as first therapy. Studies of 1L BTKi-BCL2i build upon data reporting efficacy in the R/R setting [101, 102].

However, limited available studies of 12-month IbVen combination have demonstrated similar rates of CR/CRi and EOT uMRD to those observed following VenO in CLL14. The phase II CAPTIVATE included young patients < 70 with untreated CLL, most of whom harbored adverse genetic features, pre-allocated to either fixed-duration [52] or EOT MRD-guided therapy cohorts [103] with similar rates of CR/CRi and best PB/BM uMRD rates observed. While not compared for significance, 24-month PFS rates appeared slightly lower for *TP53* aberrant versus not *TP53* aberrant CLL in the fixed-duration cohort, 84% [95% CI 63–94] versus 96% [95% CI 91–98] [52]. Use of Ib for EOT uMRD patients in MRD-cohort did not improve 12- or 30-month event-free survival rates over double-blinded placebo, and 30-month PFS rates were not superior following IbVen versus Ib for patients without EOT uMRD, see Table 1 [103]. The phase III GLOW study included older patients and/or those with comorbidities with untreated CLL comparing IbVen to CIT. Patients with CLL with known *TP53* aberrancy were excluded [34]. Higher rates of CR/CRi and best-response BM uMRD were observed following IbVen versus ChlO, with significantly longer median PFS and superior estimated 24-month PFS rates, see Table 1.

A phase II study of 1L *24-month* IbVen for patients with CLL with adverse genetic factors (92%) or older age demonstrated comparatively higher rates of CR/CRi at completion of induction (88%) [104], although BM uMRD remained similar (75% at any timepoint) [105]. Patients continued either Ib monotherapy, or more latterly IbVen, for a further 12 months if uMRD was not achieved at end of induction. For 120 patients (including 40 patients from expansion cohort), rates of 4-year PFS were and OS were 94.5% (95% CI 90.3–98.9) and 96.6% (95% CI 93.3–99.9), respectively. Rates of 4-year PFS for *TP53* aberrant CLL were 90.9% (*n* = 27) versus 95.5% (*n* = 93) [106]. It is probable that the rate of CR/CRi and PFS observed is influenced by the longer treatment duration compared with the 12-month combination in the other phase II studies.

Survival and clinical response data from the IbVen arm of the FLAIR study are awaited; however, reported MRD outcomes revealing unique differences in outcome according to IGHV mutational status corroborate with those from CAPTIVATE. Rates of BM uMRD within 24 months of treatment initiation were higher for patients with umIGHV versus mIGHV CLL (79.7% vs. 56.4%), with higher 24-month probability of uMRD, OR 3.6 (95% CI 1.59–8.15), *p* = 0.0022 [33]. Rates of both PB and BM uMRD were also higher for umIGHV versus mIGHV CLL in CAPTIVATE FD. In the latter study, no clear difference in 24-month PFS was observed by IGHV mutational status, umIGHV 93% (95% CI (93–97) versus mIGHV 97% (95% CI 88–99) [52]. The postulated mechanism is greater dependence on BCR signaling for umIGHV CLL, however as yet no differences in survival outcomes have been demonstrated.

Safety data following IbVen reported in the GLOW and CAPTIVATE studies demonstrate similar key hematological toxicities to VenO. Incidence of grade ≥ 3 neutropenia and thrombocytopenia following IbVen were lower compared with ChlO, however rates of grade ≥ 3 infection and grade ≥ 3 febrile neutropenia remained low, see Table 2 [34]. Fatal infections were seen in both arms (1.9% IbVen vs 1.0% ChlO). Similar incidence of grade ≥ 3 neutropenia (33%), with low rates of grade ≥ 3 infection (8%) and febrile neutropenia (0.6%) were observed in CAPTIVATE fixed-duration cohort [52]. Noting the limits of comparison, the incidence of grade 3–4 neutropenia, febrile neutropenia, and thrombocytopenia appear similar if not marginally higher in the comorbid population treated with VenO in CLL14, see Table 2 [7]. Incidence of any-grade diarrhea were similar between GLOW and CAPTIVATE (50.9% and 62%, respectively); the vast majority of diarrhea reported in GLOW were low-grade single events.

However, as anticipated by pre-existing safety data from trials of BTKi, there have been significant incidence of cardiac adverse events reported with 12-months IbVen in both younger and older patients. While there were similarities in incidence of hypertension between GLOW (older patients) and CAPTIVATE (young patients) studies, incidence of atrial fibrillation and sudden cardiac death appear significantly higher in older patients. Despite high rates of pre-existing hypertension in GLOW IbVen-treated patients, incidence of grade ≥ 3 hypertension was 7.5% compared with 6% in CAPTIVATE. However, reported incidences of any-grade (14.2% vs. 4%) and grade ≥ 3 (6.6% vs. 1%) atrial fibrillation appear considerably higher in GLOW compared with CAPTIVATE. In GLOW, seven (6.6%) treatment-emergent deaths, including four sudden cardiac deaths, occurred during IbVen treatment [34]. All patients who suffered sudden cardiac death had CIRS ≥ 10 and/or ECOG performance status score of 2. In CAPTIVATE approximately half of patient withdrawals/deaths during treatment in fixed-duration cohort occurred during the Ib lead-in including one sudden death (0.6%) [52]. A further cardiac death was reported during cycle 32 of Ib monotherapy in the MRD-guided cohort [103].

A theoretical concern with use of upfront BTKi-BCL2i combination therapy is acquisition of resistance mechanisms to one or both agents. Although most patients are likely to achieve deep remissions with BTKi-BCL2i, those who do not will have few effective treatment options available. Treatment options for double-refractory disease are presently limited, and overall survival is poor [107–109]. Mechanisms driving resistance to novel therapies include mutations within genes coding for target protein drug-binding sites, e.g., BTK and BCL2 or down-stream proteins, e.g., PLCγ2. There are limited data describing incidence of resistance mutations following time-limited combination therapy. Thirteen patients in CAPTIVATE fixed-duration cohort were assessed by NGS at baseline and completion of therapy without dynamic *BTK*, *BCL2*, nor *PLCγ2* mutations observed [52]. Secondary resistance mechanisms occurring within cell signaling pathways and by metabolic changes are less easily assessed in the clinic. With sequential selection pressure, subclonal changes by class-specific mutations may occur [110]. However, even time-limited simultaneous exposure to BTKi and BCL2i may theoretically incur acquisition of dynamic resistance mechanisms to both classes within the same subclone. Longer combined treatment or re-treatment with the same combination would theoretically increase this risk. However, these hypotheses are yet to be substantiated by pre-clinical work or clinical studies.

In the absence of long-term survival follow-up, there are no compelling efficacy data to support routine use of BTKi-BCL2i over VenO as first time-limited therapy for CLL. While response rates may be augmented by longer combination therapy, the theoretical risk of acquisition of resistance to both effective classes of therapy increases with cumulative exposure, and is concerning given the lack of data describing effective salvage therapy post-BTKi-BCL2i combination relapse. Early survival follow-up suggests that BTKi-BCL2i retains efficacy for *TP53* aberrant (and possibly umIGHV) CLL; the major utility of upfront BTKi-BCL2i combination may be for younger patients with high-risk disease, accepting heightened risks of hypertension and low but not insignificant cardiac arrhythmia. This combination should be used with caution in elderly patients due to significant incidence of cardiac adverse events including sudden cardiac death. Importantly, further studies and long-term follow-up of BTKi-BCL2i will enhance of our understanding of potential utility. Given the longer median-time to atrial fibrillation with Acala as continuous monotherapy [59], Acala (or Zanu) may be preferred partner to BCL2i for time-limited therapies.

## Is BTKi-BCL2i-antiCD20 triplet preferable to Ven-O?

As extension of the rationale for fixed-duration upfront BTKi-BCL2i, combining all effective agents as first therapy may conceptually result in deeper remissions with potentially improved survival outcomes. Triplet BTKi-BCL2i-antiCD20 therapy is under evaluation in several phase II, and III (ACE-CL-311), studies. While the CLL2-GIVe study evaluated the IVenO combination in patients with untreated CLL exhibiting *TP53* aberrancy only, this combination has also been studied for younger patients without *TP53*-aberrant disease (GAIA/CLL13) [25, 111].

Short follow-up of phase II studies of BTKi-BCL2i-antiCD20 triplets demonstrate impressive survival outcomes with trends to higher rates of uMRD yet similar rates of CR/CRi compared with VenO. IbVenO demonstrated 28% rate of CR with PB and BM EOT (post-C12 of triplet therapy) uMRD for patients with TN (*n* = 25) and R/R (*n* = 25) CLL (median age 59 years) as primary endpoint [112], with CR/CRi rates irrespective of MRD of 32%. The 3-year estimated PFS was similar at 95% (95% CI 72–99) for TN patients and 95% (95% CI 65–99) for R/R patients [113]. For patients with TN CLL (median age 63 years, *n* = 68), AvenO demonstrated 43% (24/59 evaluable) uMRD CR rate triplet discontinuation (C16 or C26) [114, 115] with uMRD achieved for 86% at C16 (in both PB and BM). Of patients with *TP53*-aberrant CLL (*n* = 31) [115], 45% achieved (13/29) uMRD CR rate at C16, and 86% PB and 83% BM uMRD. After median 35 months follow-up, 93% of patients have not experienced relapse/progression. Peripheral blood and BM uMRD were achieved by 89% of patients (33/37) after a median 25.8 months with CR/CRi rates of 49% following ZVenO (*n* = 39, five with *TP53* aberrancy, median age 62 years) [116, 117].

These studies demonstrate also demonstrate a trend to greater hematological toxicity with triplets than observed with VenO. Hematological AEs were similar for both TN and R/R patients following IbVenO, tending to occur early; any-grade/grade 3–4 neutropenia occurred for 94%/66% patients although one event (2%) of febrile neutropenia only was noted. Any-grade/grade 3–4 thrombocytopenia occurred for 90%/34% of patients. In addition, high incidence of any-grade/grade 3–4 hypertension were reported, 82%/38%, respectively, and 10% of patients experienced atrial fibrillation [112, 113]. Similar rates of any-grade/grade 3–4 neutropenia (75%/37%) and any-grade/grade 3–4 thrombocytopenia (73%/28%) were observed following AvenO [114], however with comparatively lower all-grade hypertension (27%) and atrial fibrillation (2.9%) observed despite longer fixed-duration therapy. The incidence of any-grade/grade ≥ 3 neutropenia and any-grade thrombocytopenia were 51%/18% and 59%, respectively, following ZVenO [116].

Both phase III trials with triplet combinations have excluded patients with *TP53* aberrancy [9, 118]. In GAIA/CLL13, venetoclax combinations VenO (*n* = 229) and IbVenO (*n* = 231) demonstrated similarly high rates of best PB uMRD (86.5% [VenO] vs. 92.2% [IbVenO]) [9] and 3-year PFS (87.7% vs. 90.5%) [25]. Although not compared for significance, three-year PFS rates following IbVenO were similar by IGHV mutation status (umIGHV 86.6% vs. mIGHV 96.0%) [10]. The authors concluded that no important differences in hematological adverse events were observed between all four arms of the study (VenO, IbVenO, VenR, CIT), with exception of incidence of grade 3–4 infections occurring for 21.2% of IbVenO treated patients compared with 13.2% in VenO arm [10]. Data from the combination arms of ACE-CL-311 (CLL-092) for CLL are yet to be reported [118].

Overall, all evaluated BTKi-BCL2-antiCD20 triplets demonstrate efficacy in short follow-up for unselected patients with CLL and those with *TP53* aberrancy. Despite augmented rates of uMRD compared with VenO, rates of CR/CRi remain < 50%. The signal for significant hematological toxicity is notable; G-CSF was required for 11% and 23% of patients receiving AVenO and ZVenO, respectively. Within limits of comparison, AVenO had similar hematological toxicity profile to IbVenO but improved cardiac toxicity, while ZVenO appeared to have slightly better hematological toxicity. Phase III data suggest seemingly minimal difference in hematological toxicity between IbVenO as compared with VenO, but greater incidence of grade 3–4 infection. While not formally compared for statistical significance, with short follow-up no clinically meaningful difference in PFS was observed between IbVenO and VenO arms.

The role of combination triplet therapy beyond pre-clinical rationale is currently uncertain in view of available data. Further phase III data is required to understand relative safety and efficacy of these combinations compared with sequenced standard of care novel therapies. Use of triplet combinations raises the same concerns about acquisition of resistance as for doublets in “If pursuing time-limited therapy, is VenO or Ven-BTKi preferred?” section, although no available data address these concerns. We suggest that it is currently difficult to justify routine use of triplet combinations over VenO given the equivalent efficacy of IbVenO in available comparative data and due to potential for significant hematological toxicity.

## Which are optimal therapies for patients with CLL exhibiting *TP53* aberrancy?

The survival outcomes for patients with CLL harboring either del(17p), *TP53* mutations, or commonly both, have traditionally been poor. Due to poor outcomes following CIT regimens which have been frequently used as comparator arms in studies, patients with any features of *TP53* aberrancy or those with known high (e.g., > 20%) tumor fractions harboring del(17p) and/or *TP53* mutations have often been excluded from key head-to-head studies using novel therapies. Recommendations for novel therapies for patients with *TP53* aberrant CLL therefore largely derive from studies including limited numbers of such patients or non-comparative data. Enrolment of patients with *TP53* aberrant CLL in clinical trials is extremely important for both patient access to potentially effective novel small molecules and increasing understanding of outcomes for this key subgroup.

Although early studies of Ib for R/R CLL suggested that *TP53* aberrancy may still portend inferior survival [119–121], in contemporaneous 1L studies outcomes for patients with *TP53*-aberrant CLL following BTKi are similar to those for patients with *TP53* wild type disease at least with available follow-up. Two phase II studies confirm meaningful disease responses in *TP53* aberrant CLL following 1L Ib; estimated 6-year PFS 60–61% [122, 123]. A pooled analysis of *TP53* aberrant CLL in key 1L Ib studies demonstrated 4-year PFS rates of 79% [28]. Final analysis of the iLLUMINATE study demonstrated no significant difference in PFS observed between patients with (*n* = 18) and without *TP53* aberrancy; HR 0.93 (95% CI 0.32–2.69), *p* = 0.895 [3]. These results are corroborated by those of ALLIANCE where no difference in PFS by *TP53* mutation status was observed in Ib-therapy arms; HR 0.99 (95% CI 0.51–1.91), *p* = 0.98 [5].

No direct comparisons of 1L Acala or Zanu by the presence or absence of *TP53* aberrancy have been reported, however both agents demonstrate efficacy irrespective of *TP53* function. Frontline Acala ± Obi demonstrated similar 4-year PFS for both patients with TP53-aberrant CLL compared with all unselected patients; 87.0% [AO] and 77.9% [A] overall, and 74.8% [AO] and 76.2% [A] for *TP53* aberrancy [6], and was shown to be as efficacious for *TP53* aberrant CLL in the R/R setting (ELEVATE RR) [59]. For patients with CLL exhibiting del(17p), continuous zanubrutinib monotherapy demonstrated ORR of 94.5% with 18-month PFS rate of 88.6% (95% CI 79.0–94.0) in the non-randomized arm of SEQUOIA [124]. Interim analyses of ALPINE study demonstrated the PFS superiority of zanubrutinib versus Ib for unselected patients with R/R CLL, which was sustained in all major sub-groups including those with *TP53* aberrancy, HR 0.53 (95% CI 0.31–0.88) [63].

Fewer phase III data describing the efficacy of BCL2i combinations as first treatment for *TP53*-aberrant CLL are available, however VenO demonstrates efficacy in this subgroup. In CLL14, high rates of uMRD and superior PFS and TTNT following VenO compared with ChlO seen overall were retained for patients with *TP53* aberrant CLL [7, 125]. While estimated 48-month PFS rates for VenO-treated *TP53* aberrant CLL were 54.2%, the presence of del(17p), along with high disease burden, were associated with inferior PFS on multivariate analysis [8]. These findings corroborate those observed for patients with R/R *TP53* aberrant CLL in MURANO at 5-year follow-up; both *TP53* disruption and/or genomic complexity were associated with lower EOT uMRD rates, inferior PFS, and OS following 24-month VenR [126]. The CLL13/GAIA study assessing the efficacy of three combinations of Ven against CIT (VenO, VenR, IbVenO) excluded *TP53* aberrant patients [9].

The efficacy of BTKi-BCL2i combinations are reported in short follow-up of phase II studies only, however demonstrate impressive CR and uMRD rates in *TP53* aberrant CLL. CLL2-GIVe evaluated the triplet combination IbVenO (IbVenO for 6 cycles, IbVen for further six cycles then MRD-guided duration of Ib monotherapy) for 41 patients with *TP53* aberrant CLL; ORR/CR + CRi and PB uMRD rates were 100%/58.5% and 78% respectively, and PFS and OS rates were both 95.1% at 24-month follow-up [111]. CAPTIVATE fixed-duration cohort evaluated 12 months of IbVen after 3 months of Ib lead-in in younger patients with CLL. Rates of CR (56% vs. 56%) and PB uMRD (81% vs. 75%) were similar for patients with CLL with (*n* = 27) or without (*n* = 129) TP53 aberrancy, although 24-month PFS rates appeared slightly inferior (84% [95% CI 63–94] vs. 96% [95% CI 91–98]) [52]. Without comparison for significance, similarly slightly lower 3-year PFS rates for *TP53* aberrant CLL (93% vs. 86%) in another phase II study evaluating 12-months of IbVen. While the GLOW study which compared the efficacy of IbVen to ChlO excluded CLL with known TP53 aberrancy, five of seven unfit/older patients with CLL exhibiting centrally-tested TP53 aberrancy treated with IbVen remained MRD negative at month 18 following completion of treatment [34].

Overall, although small numbers of patients with *TP53* aberrant CLL are included in pivotal studies of novel therapies, there are likely several effective options for this high-risk group. While the presence of *TP53* aberrancy retains adverse prognostic impact following VenO, within the limitations of available comparisons, survival outcomes are similar following BTKi and IbVen ± Obi, agnostic of *TP53* function. Zanubrutinib demonstrates superior efficacy to Ib for *TP53* aberrant CLL in the R/R setting. Broader considerations concerning selection of therapy are identical to those for patients without *TP53* aberrancy and are discussed in other sections of this review. Inclusion and enrolment of patients with *TP53* aberrant CLL patients in appropriate clinical trials remains essential for optimal drug access and growth of pre-existing data.

## Conclusions

We as physicians, and our patients with CLL, are now fortunate to have numerous established and emerging therapies for 1L treatment. With multiple options, we face challenges in optimal selection of first therapy for each patient. Clinicians need to consider treatment efficacy and tolerance in the context of disease genetic risk, patient comorbidities, and treatment goals. It is also important to consider available data supporting optimal treatment sequencing in the event of therapeutic failure.

For unselected patients of any age, we generally recommend VenO as first therapy. For those patients at considerable risk of tumor lysis or those with *TP53* aberrant disease, a BTKi may be preferred. Caution with BTKi should be exercised for older patients and/or those with pre-existing cardiac comorbidities in view of cardiac adverse events observed including sudden death, however the relative risks are likely reduced with Acala or Zanu. The merits of combination BTKi-based combination therapy, either with anti-CD20 mAb or Ven or both, have been partly evaluated; it is important to consider the potential for increase in important toxicities and acquisition of resistance mechanisms. The addition of Ritux to Ib does not confer PFS benefit, while adding Obi to Acala may improve PFS. While BTKi-BCL2i combinations may be principally considered for young patients with *TP53* aberrant disease, it is not clear whether doublets or triplets (BTKi-BCL2i ± anti-CD20 mAb) are superior to BTKi monotherapy. The role of CIT has diminished significantly, although FCR may be offered to fit patients with mutated IGHV CLL who seek long-term remission.

## Data Availability

Not applicable.

## Abbreviations

Acala: Acalabrutinib
ACDD: Antibody-dependent cellular cytotoxicity
AF: Atrial fibrillation
AO: Acalabrutinib-obinutuzumab
AVenO: Acalabrutinib-venetoclax-obinutuzumab
BCL2(i): B-cell lymphoma-2 (inhibitor)
BCLXL: B-cell lymphoma-extra large
BM: Bone marrow
BR: Bendamustine-rituximab
BTK(i): Bruton’s tyrosine kinase (inhibitor)
CDC: Complement-dependent cytotoxicity
ChlO: Chlorambucil-obinutuzumab
CIRS: Cumulative illness rating scale
CIT: Chemoimmunotherapy
CLL: Chronic lymphocytic leukaemia
CR/CRi: Complete response/complete response with incomplete haematological recovery
ECOG: Eastern Cooperative Oncology Group
EGFR: Epidermal growth factor receptor
EOT: End of treatment
ESMO: European Society of Medical Oncology
FC: Fludarabine-cyclophosphamide
FCR: Fludarabine-cyclophosphamide-rituximab
HR: Hazard ratio
Ib: Ibrutinib
IbO: Ibrutinib-obinutuzumab
IbR: Ibrutinib-rituximab
IbVen: Ibrutinib-venetoclax
IbVenO: Ibrutinib-venetoclax-obinutuzumab
IGHV: Immunoglobulin heavy chain variable region gene
ITK: IL2-inducible T-cell kinase
MAb: Monoclonal antibody
MCL1: Myeloid leukaemia cell differentiation protein
mIGHV: Mutated immunoglobulin heavy chain variable region gene
NCCN: National Comprehensive Cancer Network
NGS: Next-generation sequencing
PFS: Progression-free survival
PB: Peripheral blood
Obi: Obinutuzumab
ORR: Overall response rate
OS: Overall survival
Ritux: Rituximab
R/R: Relapsed/refractory
TEC: Tec protein tyrosine kinase
TLS: Tumor lysis syndrome
TN: Treatment-naïve
TTNT: Time to next treatment
Ven: Venetoclax
VenO: Venetoclax-obinutuzumab
VenR: Venetoclax-rituximab
umIGHV: Unmutated immunoglobulin heavy chain variable region gene
(u)MRD: (Undetectable) measurable residual disease
Zanu: Zanubrutinib
ZVenO: Zanubrutinib-venetoclax-obinutuzumab
